# Oral Metronomic Therapy: An Effective Palliative Treatment Option for Epithelial Ovarian Cancer

**DOI:** 10.7759/cureus.73171

**Published:** 2024-11-06

**Authors:** Nandini Devi R, Abhilash Menon, Praveen K Shenoy, Manuprasad Avaronnan, Sherin Shahana, Allwin George

**Affiliations:** 1 Department of Clinical Hematology and Medical Oncology, Malabar Cancer Centre – Post Graduate Institute of Oncology Sciences and Research, Thalassery, IND; 2 Department of Medical Oncology, Aster MIMS Hospital, Kannur, IND; 3 Department of Medical Oncology, AKG Hospitals, Talap, IND

**Keywords:** low- and middle-income country (lmic), oral metronomic therapy, ovarian cancer, palliative treatment, resource limited countries

## Abstract

Introduction

The majority of the patients with advanced-stage epithelial ovarian cancer relapse within three years of standard first-line treatment. Access to novel therapies like poly ADP-ribose polymerase (PARP) inhibitors and antiangiogenic agents is limited in low- and middle-income countries. Oral metronomic therapy (OMT) may be an effective alternative treatment option in resource-limited settings.

Materials and methods

A retrospective study was conducted among patients with epithelial ovarian cancer who received OMT. OMT consisted of cyclophosphamide 50mg per day and tamoxifen 40mg per day which was administered continuously until unacceptable toxicity or progression was reached. OMT was given to patients with relapsed ovarian cancer and to those newly diagnosed ovarian cancer patients who were unfit for cytoreductive surgery and/or standard first-line intravenous platinum-based chemotherapy. All patients who received OMT at our center between 01-01-2017 and 31-12-2021 were included for analysis. Relevant details, as needed for the study, were collected from medical records. SPSS version 24 (IBM Corp., Armonk, NY) was used for statistical analysis.

Results

A total of 61 patients received OMT during the study period. The median age at diagnosis of ovarian cancer was 59 years. The median age at the start of OMT was 61 years. The majority (n= 37, 60.7%) had high-grade serous carcinoma subtype. A total of 52 patients received OMT in the relapse setting while nine received it in the first-line setting. Among relapsed ovarian cancer patients, 13 patients (21.3%) experienced platinum-sensitive relapse, 16 patients (26.2%) experienced partial platinum-sensitive relapse, and 23 patients (37.7%) experienced platinum-resistant disease. The mean duration of treatment with OMT was 7.8 months. The clinical benefit rate was 59% and the overall response rate was 22.9%. Ca-125 response was seen in 40.4% of patients. The median PFS for the overall group was 6.7 months. Median PFS was longer in patients with Ca-125 response and platinum-sensitive relapse. Only five patients (8.1%) had significant toxicity.

Conclusion

OMT is a safe and effective palliative treatment option for epithelial ovarian cancer, worth consideration in low- and middle-income countries.

## Introduction

Ovarian cancer is the eighth most common cancer among female patients globally [1]. But in most Indian cancer registries, it ranks as the third most common cancer and is a leading cause of cancer-related death among female patients [2-3]. The majority of the patients present with advanced-stage disease and more than three-fourths of them recur within three years of standard first-line treatment [4]. The current treatment of relapsed ovarian cancer includes secondary cytoreductive surgery, chemotherapeutic agents, and targeted therapy including poly (ADP-ribose) polymerase (PARP) inhibitors and anti-angiogenic agents [3]. Novel agents like mirvetuximab soravtansine have also been developed [5]. However, in low- and middle-income countries, these therapies are not affordable to the majority of the patients [6]. Hence, sequential administration of various chemotherapeutic agents remains the mainstay of palliative systemic treatment for relapsed epithelial ovarian cancer. Metronomic chemotherapy is an attractive therapeutic option in these resource-limited settings [7]. 

Metronomic chemotherapy refers to the continuous administration of lower doses of chemotherapeutic agents which can produce sustainable and active plasma levels of the chemotherapy drug, without causing added toxicity [8]. Oral metronomic cyclophosphamide has been used for the treatment of advanced ovarian cancer even before the advent of platinum compounds [9]. Its objective response rates varied from 20-45% in different series and demonstrated a favorable toxicity profile [10-11]. Metronomic cyclophosphamide has also been used in combination with other chemotherapeutic agents like etoposide and topotecan, and anti-angiogenic agents such as bevacizumab and pazopanib. These combination therapies also have shown good clinical activity, especially in platinum-resistant cases [12-14].

Apart from chemotherapeutic agents, hormonal agents like tamoxifen have also been tried in the treatment of relapsed ovarian cancer for more than a decade now. Tamoxifen has been shown to have response rates of up to 56% and is cost-effective and better tolerated than chemotherapy or anti-angiogenic agents [15-17].* *Similar studies from LMICs using a combination of oral cyclophosphamide, etoposide, and tamoxifen have shown overall response rates between 15% and 60% and median progression-free survival between three to eight months. However, this combination caused significant (grade 3 or more) cytopenia in 25-45% of patients [18-20]. At our center, oral metronomic therapy (OMT) involving a combination of two drugs - cyclophosphamide and tamoxifen - was given to patients with relapsed ovarian cancer and to newly diagnosed ovarian cancer patients who were unfit for cytoreductive surgery and/or standard first-line intravenous platinum-based chemotherapy. In this study, we describe the clinical outcomes and toxicity profile of OMT.

## Materials and methods

This was a retrospective study involving patients with epithelial ovarian cancer who received OMT at the Department of Clinical Hematology and Medical Oncology at the Malabar Cancer Centre in Thalassery, Kerala, India. OMT consists of a continuous combination of tamoxifen (40 mg/day) and cyclophosphamide (50 mg/day), administered until disease progression or the onset of unacceptable toxicity. The brands of cyclophosphamide and tamoxifen used depended on what was available in our in-house pharmacy. In our center, OMT was given to patients with relapsed ovarian cancer and to newly diagnosed ovarian cancer patients who were unfit for cytoreductive surgery and/or standard first-line intravenous platinum-based chemotherapy. Newly diagnosed ovarian cancer patients were considered unfit for standard treatment if they had an Eastern Cooperative Oncology Group (ECOG) performance status of 3 or more, as assessed by two medical oncologists, and were also deemed unfit for cytoreductive surgery by an onco-surgeon. These patients were given the option of a trial of intravenous chemotherapy (either single-agent carboplatin or weekly paclitaxel-carboplatin) and re-assessment for continuation of standard chemotherapy or cytoreductive surgery by treating oncologists. If they were not willing to undergo intravenous chemotherapy, they were started on OMT. The palliative goal of treatment was explicitly explained to them. All patients who received OMT between January 1, 2017, and December 31, 2021, were included in this study. Those with incomplete medical records were excluded. The objective of this study was to assess the clinical outcomes and toxicity profile of this treatment. Clinical outcomes were measured in terms of clinical benefit rate, overall response rate, and progression-free survival as defined below.

The details regarding patient demographics, diagnostic and staging workup, treatment, follow-up, relapse, and observed toxicities were collected from the medical records. All patients underwent baseline complete blood counts, serum biochemistry including Ca-125, and contrast-enhanced CT scan of the thorax, abdomen, and pelvis before the start of OMT. While on OMT, patients underwent clinical assessment for tolerance/ toxicity every month and Serum Ca-125 level assessment every two to three months. After an initial three to four months of therapy, all patients underwent response assessment with a contrast-enhanced CT scan of the thorax, abdomen, and pelvis. For patients who had stable disease or better, OMT was continued till progression or unacceptable toxicity. Thereafter CT scan was done only when there was clinical suspicion of progression. If the patient progressed on OMT, the next line of systemic therapy or best supportive care was planned according to their performance status, prior therapies received, comorbidities, and access to targeted therapies.

This study was approved by the Institutional Review Board (1616/IRB-SRC/13/11-11-2023/2) and conformed to the Declaration of Helsinki.

The following operational definitions were used in this study. Ca-125 response was defined as at least a 50% reduction in Ca-125 levels from a pretreatment value. Radiological response assessments like complete response (CR), partial response (PR), stable disease (SD), and progressive disease (PD) were done according to Response Evaluation Criteria In Solid Tumors (RECIST) version 1.1 (https://recist.eortc.org/recist-1-1-2). Progression-free survival (PFS) was calculated from the start date of OMT to the earliest occurrence of radiological progression, death, or last follow-up. The overall response rate (ORR) was calculated as the percentage of patients who achieved complete response or partial response with OMT. Clinical benefit rate (CBR) was calculated as the percentage of patients who achieved at least stable disease with OMT (including those who experienced CR, PR, or SD). Toxicity was graded according to National Cancer Institute-Common Terminology Criteria for Adverse Events (NCI-CTCAE) version 4.03 (https://ctep.cancer.gov/protocoldevelopment/electronic_applications/ctc.htm).

Statistical methods

Since this was a retrospective study, sample size calculation was not done. All patients who received OMT during the study period were included. Statistical analysis was done using the Statistical Package for the Social Sciences (SPSS) version 24 (IBM Corp., Armonk, NY, USA). Categorical variables like stage, ECOG PS, histological subtype, platinum sensitivity status, and treatment details were expressed in percentages. Continuous variables like age and survival outcome were summarized using mean with standard deviation or median with range based on normality. Progression-free survival was calculated by the Kaplan-Meier method. Log-rank test was used to assess the association between known prognostic variables and progression-free survival. Cox regression analysis was used for multivariate analysis. A p-value <0.05 was considered significant.

## Results

Among 802 ovarian cancer patients treated between 2017 to 2021, 61 patients had received OMT. The median age at diagnosis of ovarian cancer was 59 years (range 35-77 years) and the median age at the start of OMT was 61 years (range 36-78 years). Baseline characteristics are given in Table 1. The majority of patients (n=49, 80.3%) had an Eastern Cooperative Oncology Group Performance Status (ECOG PS) of 2. Around 10 (16.3%) patients were ECOG PS-3. The high-grade serous carcinoma subtype was the most common histological subtype in 37 (60.7%) patients. Baseline serum Ca-125 levels were elevated in 52 (85.2%) patients. A total of 52 patients (85.2%) received OMT in the relapse setting while nine (14.8%) received it in the first-line setting. Among patients with relapsed ovarian cancer, 13 (21.3%) patients had platinum-sensitive relapse, 16 (26.2%) patients had partially platinum-sensitive relapse, and 23 (37.7%) patients had platinum-resistant disease. All nine (14.8%) patients who received OMT as first-line treatment were above 70 years of age and were unfit for intravenous chemotherapy or cytoreductive surgery. About 16 (26.2%) patients had symptomatic ascites requiring therapeutic paracentesis before the start of OMT. 

**Table 1:** Baseline characteristics of patients who received OMT OMT, oral metronomic therapy; ECOG PS, Eastern Co-operative Oncology Group Performance Status; NOS, not otherwise specified

Baseline characteristic	N (%)
Median age at diagnosis (in years)	59
Median age at start of OMT (in years)	61
ECOG PS at the start of OMT	
ECOG PS-2	49 (80.3)
ECOG PS-3	10 (16.4)
ECOG PS not available	2 (3.2)
Stage at the start of OMT	
Stage 3	20 (32.8)
Stage 4	41 (67.2)
Histological subtype	
High-grade serous carcinoma	37 (60.7)
Low-grade serous carcinoma	4 (6.6)
Clear cell adenocarcinoma	1 (1.6)
Mucinous adenocarcinoma	1 (1.6)
Endometroid adenocarcinoma	0
Carcinoma, undifferentiated, NOS	18 (29.5)
Presence of ascites requiring therapeutic paracentesis at the start of OMT	16 (26.2)
Peritoneal disease at the start of OMT	46 (75.4)
Prior history of cytoreductive surgery	31 (50.8)
Platinum sensitivity status at the start of OMT	
Platinum-sensitive relapse	13 (21.3)
Partially platinum-sensitive relapse	16 (26.2)
Platinum-resistant relapse	23 (37.7)

Clinical outcomes

The mean duration of treatment with OMT was 7.8 months. Treatment details are shown in Table 2. At the time of this analysis, six patients (9.8%) were still continuing OMT. Symptomatic relief was documented in 34 (55.7%) patients. Among the 16 patients with symptomatic ascites at baseline, the frequency of therapeutic paracentesis reduced in 13 (81.1%) after starting OMT. Ca-125 response was seen in 21 (40.4%) patients. Only one patient achieved a complete response. The majority had stable disease (n=22, 36.1%). Clinical benefit was seen in 36(59%) patients. A total of 14 patients (22.9%) had at least partial response and the overall response rate was 22.9%; 19 patients (31.1%) had refractory disease.

**Table 2:** Treatment details of patients who received OMT

Variable	N (%)
Treatment setting	
First-line treatment	9 (14.8)
Second-line treatment	20 (32.8)
Third-line treatment	15 (24.8)
Fourth-line and beyond	17 (27.8)
Maximum response documented while on OMT	
Complete response	1 (1.6)
Partial response	13 (21.3)
Stable disease	22 (36.1)
Progressive disease	19 (31.1)
Not available	6 (9.8)
Proportion of patients continuing OMT at the time of analysis	6 (9.8)
Reason for discontinuation of OMT (n=55)	
Disease progression	44 (72.1)
Toxicity	3 (4.9)
Logistic issues	6 (9.8)
Not available	2 (3.2)
Post-OMT treatment (n=55)	
Best supportive care	22 (36.1)
Oral etoposide	6 (9.8)
Intravenous chemotherapy	24 (39.3)
Not available	3 (4.9)

Survival outcomes

The median duration of follow-up was 27 months. The median PFS was 6.7 months. The median PFS of patients with platinum-sensitive relapsed ovarian cancer was significantly longer than those with platinum-resistant disease (9.8 months vs 4.2 months, p=0.015). Similarly, the median PFS of patients with Ca-125 response was significantly longer compared to those without Ca-125 response (10.7 months vs 3.5 months, p<0.01). The median PFS of patients who received OMT as first-line treatment was 9.8 months. The median PFS of patients with ECOG PS-3 was 2.1 months. The factors affecting PFS are shown in Table 3. In univariate analysis, platinum sensitivity status at the start of OMT, and Ca-125 response were found to be significant predictors of progression-free survival. However, in multivariate analysis, only the Ca-125 response was found to be predictive of progression-free survival. 

**Table 3:** Univariate analysis of factors affecting progression-free survival *statistically significant association; p-value < 0.05 is considered significant

Factors affecting PFS	Median PFS in months	p-value
Platinum sensitivity at start of OMT		
Platinum-sensitive	9.9	0.015*
Partially platinum-sensitive	6.4	
Platinum-resistant	4.2	
Stage at start of OMT		
Stage 3	6.9	0.833
Stage 4	4.9	
Peritoneal disease at start of OMT		
Present	6.9	0.495
Absent	9.9	
Prior cytoreductive surgery		
Present	6.4	0.986
Absent	6.9	
Ca-125 response		
Present	10.7	<0.01*
Absent	3.5	

Safety outcomes

Eight patients (13.1%) had treatment-related toxicity during OMT, of which only five patients (8.1%) had more than or equal to grade 3 toxicity and all were hematological adverse effects. Hematological toxicity was seen in a total of six (9.8%) patients. Three (4.9%) patients had neutropenia and three patients (4.9%) had thrombocytopenia. None of them had febrile neutropenia. In patients with significant cytopenia, the first dose modification to 50mg/day for 21 days every 28 days was administered. If cytopenia persists a second dose modification to 50mg/day for 14 days every 28 days was done. If cytopenia persisted after two dose modifications, OMT was discontinued permanently. None of the patients needed transfusion support. Dose modification was done in four patients (6.6%). Treatment was discontinued due to cytopenia in three patients (4.9%). Among non-hematological toxicities, one patient (1.6%) had grade 2 fatigue, and one (1.6%) patient had grade 1 nausea and vomiting which was managed with anti-emetics. A comparison of our study with other OMT regimens is given in Table 4 below.

**Table 4:** Different metronomic therapy regimens used for epithelial ovarian cancer treatment compared to our study OMT: oral metronomic therapy; DCR: disease control rate; CBR: clinical benefit rate; N/A: not available; ORR: objective response rate; PFS: progression-free survival; OS: overall survival *In the study by Ghosh et al., OS data was reported exclusively in years [25].

Study	OMT regimens used	ORR (%)	PFS in months	OS in months	Grade 3 or 4 toxicities encountered
Gupta et al. [19]	Combination of etoposide 50mg/day, cyclophosphamide 50mg/day, and tamoxifen 20mg/m2/day	45.8	7.97	22.3	Anemia (44%), neutropenia (36%), febrile neutropenia (12%)
Ferrandina et al. [10]	Cyclophosphamide 50mg/day	40.8	4	13	Anemia (1.8%), gastritis (1.6%), asthenia (1.6%)
Bhattacharya et al. [21]	Combination of cyclophosphamide 50mg/day, and temozolomide 20mg BD for 14 days, every 3 weeks	44.4	5.9	10.1	Febrile neutropenia (1.8%), thrombocytopenia (22%), neutropenia (20%)
Dinkic et al. [22]	Combination of cyclophosphamide 50mg/day and pazopanib 400-800mg/day	36	8.35	24.9	Transaminitis (25%)
Huang et al. [11]	Cyclophosphamide 50-100mg/day	25 (DCR, 61%)	4.29	11.26	Nausea/vomiting (6%), neutropenia (5%)
Sharma et al. [23]	Combination of etoposide 50mg/day, cyclophosphamide 50mg/day ± pazopanib 200-400mg/day ± celecoxib 200mg/day	50	8.2	38	Neutropenia (22.2%)
Gulia et al. [14]	Combination of cyclophosphamide 50mg /day for 21 days every 28 days and pazopanib 600mg/day	75	5.5	9.5	Fatigue (25%), diarrhea (15%), hand-foot syndrome (10%)
Banerjee et al. [20]	Combination of cyclophosphamide 25mg/day, etoposide 25mg/day, and tamoxifen 20mg/day	15	4	NA	N/A
Wysocki et al [24]	Combination of topotecan 1mg BD ± cyclophosphamide 50mg/day	27	3.65	NA	Anemia (25.9%), neutropenia (18.5%), transaminitis (4%),
Harsh et al. [18]	Combination of cyclophosphamide 50mg/day, etoposide 50mg/day, and tamoxifen 20mg BD/day	60	3.7	6.5	Anemia (30%), febrile neutropenia (25%), thrombocytopenia (22.5%), mucositis (22.5%)
Ghosh et al. [25]	Combination of cyclophosphamide 50mg/day and capecitabine 500mg BD/day	79	9	3 years*	Nausea/vomiting (8.3%)
Martorana et al. [26]	Cyclophosphamide 50mg/day	18.8 (DCR, 36.2%)	3.16	8.7	N/A
Our study 2024	Combination of cyclophosphamide 50mg/day and tamoxifen 40mg/day	22.9 (CBR, 59%)	6.7	NA	Thrombocytopenia (4.9%), neutropenia (3.%)

## Discussion

In our study, we found OMT to be an effective and safe palliative treatment option for patients with epithelial ovarian cancer. Patients with relapsed ovarian cancer treated with OMT had comparable progression-free survival to contemporary intravenous chemotherapeutic agents in both platinum-sensitive and resistant settings. Elderly patients who are unfit for standard first-line intravenous chemotherapy had reasonable clinical benefits with OMT. Only five patients (8.1%) had grade 3 or grade 4 toxicity. Hence OMT is a feasible palliative treatment option for patients with epithelial ovarian cancer. 

Baert et al., in their review, have shown an ORR of 16-66% for platinum-based chemotherapy and 16-35% for non-platinum-based chemotherapy in relapsed ovarian cancer patients. Also, platinum-based chemotherapy regimens had a median PFS ranging between 7 to 13 months and non-platinum chemotherapeutic agents had a median PFS between 3.5 and 9 months [27]. The clinical benefit rate and overall response rate with OMT were 59% and 23% respectively in our study. The median PFS in the overall group was 6.7 months which was comparable to the aforementioned meta-analysis. In patients with platinum-sensitive relapsed ovarian cancer, the median PFS was 9.8 months, and in platinum-resistant disease, the median PFS was 4.2 months. These results show that OMT can be considered as an alternative palliative treatment option for relapsed ovarian cancer.

In the systematic review by Baert et al., for platinum-resistant relapsed ovarian cancer, the median PFS with different non-platinum chemotherapeutic agents was between three to nine months. The chemotherapeutic agents used were weekly paclitaxel, topotecan, liposomal doxorubicin with/without trabectedin, and gemcitabine. These chemotherapeutic agents caused significant toxicities including fatigue, alopecia, hand-foot syndrome, gastrointestinal disturbances, myelosuppression, and peripheral neuropathy [27]. In our study, the median PFS of platinum-resistant relapsed ovarian cancer patients was 4.2 months and only five patients (8.1%) had grade 3 or higher toxicity. For platinum-resistant relapsed ovarian cancer patients, OMT can be an effective and safe palliative treatment option.

In the 2021 GINECO/GCIG trial, in elderly patients above 70 years of age, three chemotherapy regimens were compared (thrice-weekly single-agent carboplatin, thrice-weekly paclitaxel-carboplatin, and weekly paclitaxel-carboplatin) resulting in median PFS periods of 4.8 months, 12.5 months, and 8.3 months, respectively. Treatment-related adverse events occurred in more than 40% of all three arms of this trial [28]. In our study, among the unfit elderly patients who received OMT as first-line therapy, the median PFS was 9.8 months. Though our numbers are low, OMT seemed to be an effective palliative treatment option for frail elderly patients unfit for standard therapy.

With the addition of bevacizumab to chemotherapy, the median PFS was around 12 months in the OEANS study and 6.7 months in the AURELIA study [29-30]. With OMT, comparable results were seen in our study i.e. 9.8 months in platinum-sensitive relapse setting and 4.2 months in platinum-resistant relapse setting. In resource-limited settings, where targeted therapies like bevacizumab are not affordable, OMT may be an alternative palliative treatment option.

The results of SOLO-3 and ARIEL-4 trials demonstrated the detrimental effects of PARP inhibitors in later lines of treatment. However, in our study, more than 50% of patients received OMT as the third line of treatment or beyond and had a median PFS of 4.16 months which was noteworthy. Additionally, OMT is easily accessible and affordable for all patients compared to PARP inhibitors [7].

The various metronomic therapies (shown in Table 4) had response rates between 15% to 79%. Median PFS ranged between three to nine months. Hematological toxicities were very common during these therapies. Fatigue and gastrointestinal disturbances were the most common non-hematological toxicities. Studies utilizing the combination of oral metronomic cyclophosphamide, etoposide, and tamoxifen had overall response rates between 15% and 60% and median PFS between three to eight months. But this triple drug combination combination caused significant cytopenia in 25-45% of patients [18-20]. The clinical benefit rate and overall response rate with our two-drug OMT were comparable with the aforementioned OMT regimens. The more important observation was the lower rates of grade 3 or more toxicity in our study. The OMT regimens with three-drug combinations had higher toxicity rates compared to ours. So, our OMT regimen seems to be a relatively safe treatment option for epithelial ovarian cancer patients.

Limitations

The major limitation of our study was the inherent bias and missing data due to its retrospective nature. Additionally, we were unable to conduct an objective assessment of the quality of life parameters. Being a single-center study, the generalizability of results is limited; however, as a randomized controlled trial might not be ethically feasible in this setting, future research might need to judiciously utilize the data from retrospective studies such as ours.

## Conclusions

OMT is a safe and effective palliative treatment option for epithelial ovarian cancer, worth consideration in low- and middle-income countries. It had comparable response rates and PFS outcomes to contemporary intravenous chemotherapeutic agents in both platinum-sensitive and platinum-resistant settings. Even in frail and elderly ovarian cancer patients who are unfit for intensive treatment, OMT provides reasonable clinical benefits that are worth exploration in future prospective trials.
